# Fragranced Products and VOCs

**DOI:** 10.1289/ehp.1103497

**Published:** 2011-05

**Authors:** Madhuri Singal, Danielle Vitale, Ladd Smith

**Affiliations:** Research Institute for Fragrance Materials Inc., Woodcliff Lake, New Jersey, Email: msingal@rifm.org

In the article “Scented Products Emit a Bouquet of VOCs,” Potera (2011) gave a broad overview of the work of Steinemann et al. (2010) regarding the quantification of volatile organic compounds (VOCs) from fragranced products. Unfortunately, crucial facts were omitted about the materials cited and the use of alternative substances.

Potera (2011) quoted Steinemann et al. (2010), noting that some of the VOCs detected “are classified as toxic or hazardous by federal laws” and “a single fragrance in a product can . . . react with ozone in ambient air to form dangerous secondary pollutants.” Potera stated that limonene reacts with ozone to form formaldehyde but failed to mention that both limonene and pinene are naturally occurring materials found in citrus fruits and pine trees, respectively (Wei and Shibamoto 2007). Fragrance materials are naturally volatile; otherwise, they would not be detectable (Cometto-Muñiz et al. 1998). Langer et al. (2008) showed that exposure to limonene from peeling an orange is far greater than using limonene-scented cleaning products. These authors further showed that secondary organic pollutants formed from cleaning products exist in the lowest range of exposure and that a higher concentration of particulates is formed by peeling an orange.

Potera (2011) quoted Steinemann et al. (2010), noting that “133 unique VOCs [were] identified among 25 products”; however, not all of the 133 VOCs are used as fragrance materials. For example, the highest reported concentration of *d*-limonene was 135 mg/m^3^ (unidentified air freshener) in an experiment using conditions completely atypical of consumer use (Steinemann et al. 2010).

Although, the U.S. Environmental Protection Agency does not issue safe exposure limits, they report those from other agencies [National Institute for Occupational Safety and Health (NIOSH), Occupational Safety and Health Administration (OSHA), and American Conference of Governmental Industrial Hygienists (ACGIH)]. As of today, none of these agencies has issued a limit value for *d*-limonene. Germany (NIOSH 2005) and Sweden (International Agency for Research on Cancer 1999) have established limits for *d*-limonene of 110 mg/m^3^ and 150 mg/m^3^, respectively. Even under the adverse testing conditions reported by Steinemann et al. (2010), the *d*-limonene concentration of 135 mg/m^3^ still falls within safe exposure.

Potera (2011) cited a telephone survey by Caress and Steinemann (2009) that attributed consumer health problems to the use of scented products; however, the percentages were not in context with the total population surveyed. Of those surveyed, 19% reported unspecified health problems and 11% noted irritation, all of which were subjectively ascribed to the use of scented laundry products (Caress and Steinemann 2009). While consumer complaints should be taken seriously, one may question the investigators’ acceptance of these self-assessments in the absence of objective confirmation by medical testing.

Potera (2011) quoted Claudia Miller, who stated that “we need to find unscented alternatives ….” The fact is a variety of scented and unscented consumer products exist; thus, it is unnecessary to use potentially dangerous home mixtures, such as vinegar (acetic acid) and baking soda (sodium bicarbonate), which was recommended as a replacement for commercial cleaning products (Potera 2011). However, the safe exposure level for acetic acid, according to the ACGIH, NIOSH, and OSHA, is 25 mg/m^3^ over 8 hr (OSHA 2007), which suggests a higher toxicity than for limonene. Health effects resulting from inhalation exposure to acetic acid include respiratory irritation, coughing, headache, and dizziness (Iowa State University 2000).

In addition, symptoms include pulmonary edema, chest pain, and hypotension; in contrast, *d*-limonene has not been associated with the development of any of these symptoms. Lacking published inhalation safety information for sodium bicarbonate, NIOSH recommends using a respirator when working with the dry particulate form (Mallinckrodt Baker Inc. 2009).

Potera (2011) ended her article by quoting Claudia Miller’s statement that “the best smell is no smell.” This is a very subjective assessment and cannot be characterized as an objective, science-based conclusion supported by available data.
